# Effect of the Nurse-Led Sexual Health Discharge Program on the Sexual Function of Older Patients Undergoing Transurethral Resection of Prostate: A Randomized Controlled Trial

**DOI:** 10.3390/geriatrics5010013

**Published:** 2020-03-02

**Authors:** Ramin Bayat, Hooman Shahsavari, Soghrat Faghihzadeh, Sara Amaniyan, Mojtaba Vaismoradi

**Affiliations:** 1 Faculty of Nursing and Midwifery, Tehran University of Medical Sciences, 1419733171 Tehran, Iran; baiatramin@gmail.com; 2 Faculty of Medicine, Zanjan University of Medical Sciences, 4513956111 Zanjan, Iran; s.faghihzadeh@zums.ac.ir; 3 Nursing Care Research Center, Semnan University of Medical Sciences, 3514799442 Semnan, Iran; amanian51@yahoo.com; 4 Faculty of Nursing and Health Sciences, Nord University, 8049 Bodø, Norway; mojtaba.vaismoradi@nord.no

**Keywords:** cancer, discharge planning, nursing, older patients, education, sexual function, transurethral resection of prostate

## Abstract

**Background**: Sexual dysfunction is a complication of transurethral resection of prostate (TURP). There is a lack of knowledge of the effect of discharge programs aiming at improving sexual function in older patients undergoing TURP. **Objective**: To investigate the effect of the nurse-led sexual health discharge program on the sexual function of older patients undergoing TURP. **Methods**: This randomized controlled clinical trial was conducted on 80 older patients undergoing TURP in an urban area of Iran. Samples were selected using a convenience method and were randomly assigned into intervention and control groups (*n* = 40 in each group). The sexual health discharge program was conducted by a nurse in three sessions of 30–45 min for the intervention group. Sexual function scores were measured using the International Index of Erectile Function (IIEF) Questionnaire, one and three months after the intervention. **Results**: The intervention significantly improved erectile function (*p* = 0.044), sexual desire (*p* = 0.01), satisfaction with sexual intercourse (*p* = 0.03), overall satisfaction with sexual function (*p* = 0.01), and the general score of sexual function (*p* = 0.038), three months after the program. In the first month after the intervention, except in sexual desire (*p* = 0.028), no statistically significant effect of the program was reported (*p* > 0.05). **Conclusion**: The nurse-led sexual health discharge program led to the improvement of the sexual function of older patients undergoing TURP over time. This program can be incorporated into routine discharge programs for the promotion of well-being in older patients.

## 1. Introduction

Benign prostatic hyperplasia (BPH) as an age-related process is characterized by the hypertrophy of the prostate gland [1] and negatively influences patients’ quality of life [2]. The aging process is associated with lower urinary tract symptoms independent of the enlargement of the prostate gland [3] and affects 28% of men aged more than 70 years [4].

Transurethral resection of prostate (TURP) is one of the conventional treatments of BPH [5] and can lead to complications such as early iatrogenic stress incontinence (30%–40%), stricture (2.2%–9.8%), erectile issues (3.4%–32%), and ejaculatory dysfunction (53%–75%) [6]. Additionally, patients often feel distress with regard to their sexual function and sexuality [7]. Therefore, management of their sexual dysfunction needs multi-dimensional programs consisting of physiological, psychological, and therapeutic interventions [8]. It has been shown that a 10-week group-based cognitive-behavioral program can improve the sexual function of patients after radical prostatectomy [9]. Additionally, continuous improvements in sexual outcomes after the use of the internet-based sexual counseling program in patients with prostate cancer have been reported [10].

Planning for the discharge process and transnational care to home is an important task of healthcare professionals, especially nurses [11]. The discharge program should be started upon admission, continued to the discharge and follow up visits [12], as well as should be specialized to each patient’s needs [13]. The discharge planning program has positive effects on mental health [11] and cardiovascular health [13]. It addition, it can reduce the readmission rate of patients to the hospital [14]. However, there is a lack of evidence surrounding the effect of the discharge program run by the nurse on sexual function among older patients with prostate disorders. Therefore, this study examined the effect of the nurse-led sexual health discharge program on the sexual function of older patients undergoing TURP.

## 2. Materials and Methods

### 2.1. Study Design

**Figure 1:**
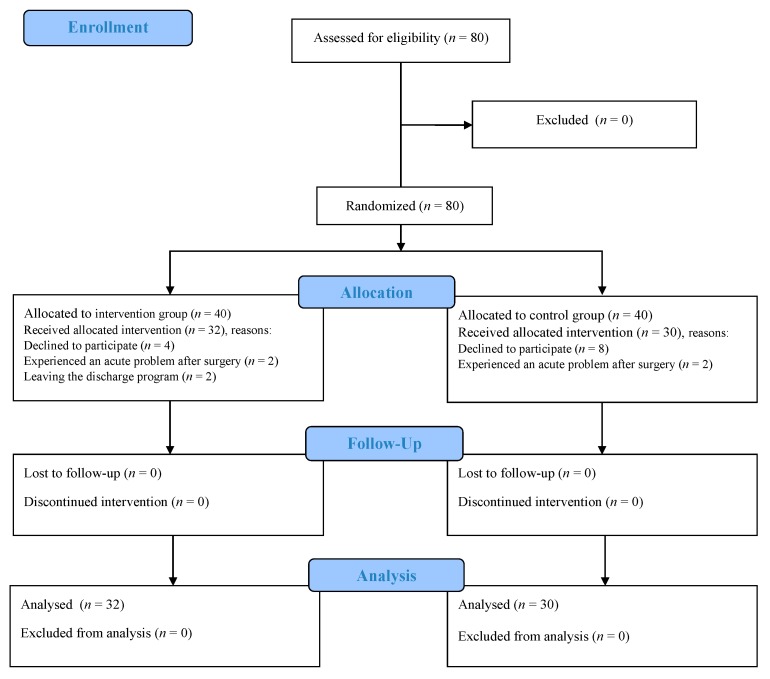
The study process according to the CONSORT flow diagram.

This randomized controlled trial with one intervention group and one control group was conducted from June 2017 to April 2018. The study process was depicted using the CONSORT flow diagram (Figure 1).

Given a 15% chance of attrition rate, α = 0.05, and a power of 0.80, the sample size was estimated 40 people in each group. Accordingly, 80 older patients undergoing TURP were selected using a convenience sampling method from the urological ward of a referral teaching hospital in an urban area of Iran. They were randomly assigned to each group via a 4:4-block randomization method by a staff nurse who was unaware of the assignments through a system of sealed envelopes. Allocation codes were written on the separate pieces of paper, placed in sealed envelopes and in an opaque box. The envelopes were removed from the box to determine the patient’s group by the staff nurse. After opening them, they were put back into the box. The sampling was continued until that the samples in both groups was accomplished. Identity of research hindered blinding of the subjects to the group assignments. However, the data analyst (SF) was unaware of the group assignments and the codes assigned to each group was disclosed after accomplishing data analysis.

### 2.2. Setting and Participants

Inclusion criteria to recruit the participants were as follows: (i) older patients aged between 60 and 85 years; (ii) being married; (iii) no history of receiving sexual health education; (iv) no hypogonadism and neurological disorders such as multiple sclerosis and spinal cord injuries; (v) no history of the pelvic floor muscle surgery; (vi) no consumption of opioids, alcohol, anti-depressants, or anti-dementia drugs; (vii) no previous psychiatric disorder; and (viii) no history of coronary artery diseases, bypass graft surgery, and current atherosclerotic diseases. Unwillingness to participate in the study, not following up to the scheduled discharge program, and the experience of an acute health problem after surgery led to exclusion from the study.

### 2.3. Measures

Data were collected using a demographic data form and the International Index of Erectile Function (IIEF) Questionnaire.

○ The demographic data form for gathering data on the older patients’ age, body mass index, education level, health insurance status, employment status, economic status, independence in the activities of daily living, and past medical history.
○ The IIEF Questionnaire was developed and validated by Rosen in 1997. It is a self-report questionnaire of the male sexual function and consists of 15 questions with a 5-point Likert scale. The minimum and maximum scores were 15 and 75, respectively and a higher score indicated a better sexual performance. Severity of erectile dysfunction was classified as follows: 6–10 (severe), 11–16 (moderate), 17–21 (mild-to-moderate), 22–25 (mild), 26–30 (no dysfunction) [15]. More details on the number of items and relating scores were presented in Table 1.

### 2.4. Validity and Reliability

For content validity, the demographic data form and the IIEF questionnaire were given to 10 specialists of nursing, reproductive health, and urology to seek their opinions. For reliability using internal consistency, the Cronbach’s alpha coefficient of the IIEF questionnaire was calculated and reported 0.97.

### 2.5. Ethical Considerations

Ethical permission was obtained from the ethics committee affiliated with university in which the second author (HS) worked (decree code: IR.TUMS.FNM.REC.1396.2300). The purpose and method of the discharge program were explained to the participants before commencing the study, and they were asked to sign the written consent form, if they were willing to take part in the study. They were assured of the possibility of withdrawal from the study without any effect on the process of care. Additionally, the older patients’ names remained confidential during the study and publication of findings. This clinical trial was registered on the website of registry of clinical trials under the code of IRCT20180917041052N1.

### 2.6. Intervention

The nurse-led sexual health discharge program was designed based on a thorough review of online literature and nursing textbooks [1,8], as well as consultation with experts in the fields of cancer nursing and urology.

Before the intervention, a demographic data form was completed and the sexual function of the older patients in the intervention and control groups was measured using the IIEF questionnaire. Next, the program was conducted for the intervention group in the urology ward by the first researcher (RB) who was a staff nurse and had required education and training regarding the pedagogical approach of sexual function during three teaching sessions as follows:

○ *The first session*: Description of the anatomy and physiology of the urinary tract and prostate gland function, definition of BPH, causes, and clinical manifestations [1,8].
○ *The second session*: Diagnosis of BPH, explanation of TURP as a treatment modality of BPH, probable complications of TURP including pain, infection, bleeding, urinary incontinence, and sexual dysfunction [1,16].
○ *The third session*: Effect of fatigue on sexual function, the importance of managing urinary incontinence for improving sexual satisfaction, role of physical activity and exercise in the recovery of sexual performance, role of pelvic floor muscle exercises in the improvement of sexual function and activity through controlling urinary incontinence [8,17,18].

The duration of each education session varied from 30 to 45 min. It was presented to the older patients individually from admission until discharge from the hospital, face to face in their rooms, and with the consideration of the older patients’ sexual health level and past knowledge. Education was discontinued in the first hours after surgery and when they had pain, bleeding and anxiety. To ensure of the effectiveness of education and the older patients’ learning, their questions were answered through holding discussions. In addition, educational materials were provided to the older patients via an educational booklet.

For followed up, the first researcher made telephone calls every two weeks to answer the patients’ questions and monitor the implementation of the discharge program at home after discharge. In addition, the researcher’s telephone number was given to the participants for necessary contacts.

The control group only received routine care consisting of brief education by staff nurses in the ward regarding discharge care. Sexual function in both groups was measured one and three months after discharge using the questionnaires.

### 2.7. Data analysis

Descriptive (frequency, percentage, mean and standard deviation) and inferential statistics (Fisher exact test, independent t-test, paired-t test, and ANOVA test) were used for data analysis. Simple linear regression was used to assess differences in sexual function between the groups before, one month and three months after the intervention. To investigate the effect of time trends, intervention, and interactions between time and groups, the repeated measures of analyses of variance were used. All statistical analyses were performed using the SPSS software version 16 for Windows (SPSS Inc., Chicago, IL, USA) and p value less than 0.05 was considered statistically significant.

## 3. Results

The mean age of the older patients in the intervention and control groups was 64.13 ± 6.23 and 67.27 ± 6.80, respectively. The groups were homogenous in terms of age, body mass index, occupation status, health insurance, economic status, independence in the activity of daily living, history of hypertension, diabetes mellitus, heart failure, smoking, use of drugs for BPH and chronic obstructive pulmonary disease (COPD) (*p* > 0.05). However, there was a statistically significant difference between the groups in the education level (*p* = 0.018) (Table 2).

Regarding the sexual function (Table 3), the mean and standard deviation of sexual performance in the intervention and control groups before the intervention were 45.12 ± 22.21 and 44.03 ± 24.34, respectively. There were no statistically significant differences between the groups in terms of sexual function categories and overall sexual function.

The mean score and standard deviation of the sexual function in the intervention and control groups one month after the intervention were reported 31.72 ± 22.38 and 21.03 ± 21.93, respectively. The program did not have any statistically significant effect on the older patients’ sexual function in this time period except on the sexual desire domain (*p* = 0.028).

Three months after the intervention, the mean scores of sexual function in the groups were reported 43.75 ± 22.62 and 29.60 ± 20.18, respectively. The assessment of sexual function between the groups three months after the intervention demonstrated that the scores of erectile function (*p* = 0.044), sexual desire (*p* = 0.01), satisfaction with sexual intercourse (*p* = 0.03), overall satisfaction (*p* = 0.01) domains, and also the overall score of sexual function (*p* = 0.038) had statistically significant differences. Therefore, the nurse-led sexual health discharge program had statistically significant effects on these domains over time. However, no statistically significant difference was found between the groups in orgasmic function (*p* = 0.331) (Table 3).

**Figure 2:**
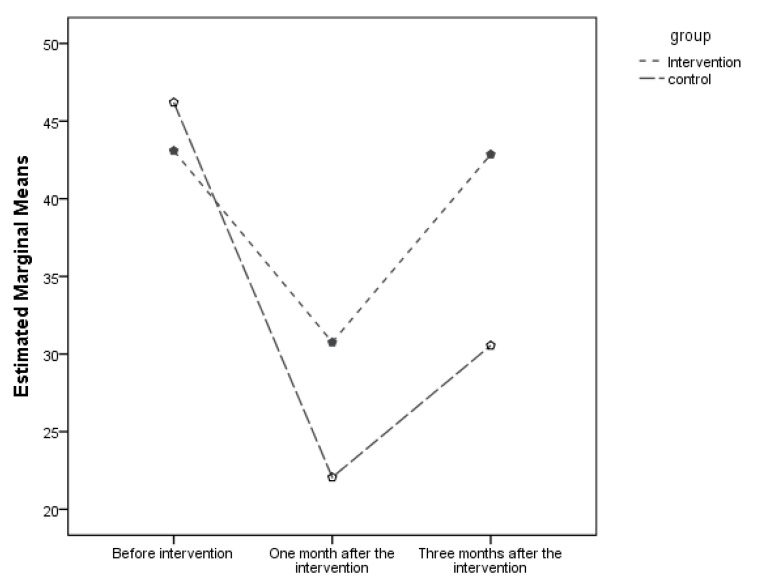
Mean scores of sexual function at different times.

To examine and compare the sexual performance of the older patients before, one month and three months after the intervention in the groups, the analysis of variance with repeated measures showed that the intervention without the effect of time had no statistically significant effect on sexual function (*p* = 0.24), but a statistically significant effect was observed in examining the interaction between time and the intervention on sexual function (*p* = 0.04). This meant that for the intervention to create a positive effect on the older patients’ sexual function, time was required and positive results were obtained over a longer period of time (Table 4; Figure 2).

## 4. Discussion

The present study aimed to evaluate the older patients’ sexual function undergoing TURP after the implementation of the nurse-led discharge program. Regarding the comparison of sexual function before and three months after the intervention, the total mean score of sexual function in the intervention group was significantly higher than that of the control group. However, at the first month after the intervention, only improvements in sexual desire were observed in the intervention group. Statistically insignificant changes on other areas could be due to the physical consequences of surgery such as the presence of urinary catheter, pain, dysuria, bleeding of the urethra, and incontinence after TURP.

Regarding the role of physical activity in the improvement of physical and psychological sexual function, our study results supported the findings of Simon et al.’s study in the USA [19]. They found that exercise had a positive correlation with the sexual function of participants. In addition, the results of the present study were in line with those of Geraerts et al.’s (2016) study in Belgium [20] investigating the effect of pelvic floor muscle exercises on erectile dysfunction and ejaculation after radical prostatectomy, indicating the improvement of sexual function after the intervention.

Considering the psychological part of the educational discharge program, our findings were supported by those of Brotto et al. (2008) in Canada [21], indicating that the psychological education intervention significantly reduced women sexual dysfunction. In another study, Lassen et al. (2013) in Canada [22] showed that nursing discharge planning based on psycho-educational interventions had positive effects on patients’ sexual function after prostatectomy.

No study was found in the literature to assess the impact of the nurse-led discharge program on the sexual function with a focus on both physical and psychological aspects in older patients undergoing TURP. This study considered various physical and psychological factors influencing the sexual health of older patients. Therefore, it had a holistic perspective toward the discharge program to cover all dimensions influencing their sexual function [23].

A limitation of this study was that the researcher had no possibility to educate the older patients’ partners and spouses with regard to sexual function. Therefore, further studies are needed to assess the status and sexual satisfaction of their spouses and partners. In addition, the small sample size and a focus on older patients one month and three months after TURP could influence the generalizability of findings to patients in other age groups and in longer follow up periods. Due to the interaction between time and intervention on sexual function, future studies with a longer intervention duration are suggested to evaluate the effect of time on sexual function.

## 5. Conclusions

This was the first randomized controlled trial on the effect of the nurse-led discharge program on the sexual function of older patients undergoing TURP with a focus on both physical and psychological aspects influencing sexual function. The results of this trial showed that a nurse-led discharge program had a positive effect on the sexual function of older patients undergoing TURP. This program can be incorporated into routine discharge planning for the promotion of well-being in older patients. Nurses are suggested to perform a need assessment and design and implement discharge programs for improving the sexual health and quality of life of older patients undergoing invasive procedures such as TURP.

## Figures and Tables

**Table 1:** Items and the scores of the International Index of Erectile Function (IIEF) Questionnaire.

Domain	Items	Score Range	Maximum Score
Erectile function	1–5, 15	0–5	30
Orgasmic function	9–10	0–5	10
Sexual desire	11–12	1–5	10
Intercourse satisfaction	6–8	0–5	15
Overall satisfaction	13–14	1–5	10

**Table 2:** Demographic characteristics of the older patients in the groups.

Variable		Mean ± Standard Deviation				Statistical Analysis/ *p*-Value
		Intervention Group		Control Group		
**Age (year)**		6.23 ± 64.13		6.80 ± 67.27		Df = 60 *t* = −1.897 *p* = 0.063
**Body mass index**		3.22 ± 24.5		2.71 ± 24.28		*p*= 0.529
		**Intervention group**		**Control group**		
		*n*	%	*n*	%	
**Education level**	Illiterate	5	15.6	5	16.7	*p* = 0.018
	Primary	7	21.9	17	56.6	
	Under diploma	7	21.9	5	16.7	
	Diploma	12	37.5	3	10	
	Academic	1	3.1	0	0	
**Employment Status**	Employee	6	18.8	5	19.7	*p* = 0.752
	Retired	22	68.7	19	60.3	
	Other	4	12.5	6	20	
**Insurance**	Yes	32	100	30	100	*p* = 0.99
	No	0	0	0	0	
**Supplementary Insurance**	Yes	32	100	28	93.3	*p* = 0.230
	No	0	0	2	6.7	
**Economic status**	Good	31	96.9	29	96.7	*p* = 0.999
	Not good	1	3.1	1	3.3	
**Dependency in daily living**	Yes	31	96.9	30	100	*p* = 0.999
	No	1	3.1	0	0	
**Hypertension**	Yes	9	28.1	6	20	*p* = 0.558
	No	23	71.9	24	80	
**Diabetes**	Yes	5	15.6	2	6.7	*p* = 0.427
	No	27	84.4	28	93.3	
**Heart failure**	Yes	3	9.4	2	6.7	*p* = 0.999
	No	29	90.6	28	93.3	
**Smoking habit**	Yes	5	15.6	3	10	*p* = 0.709
	No	27	84.4	27	90	
**Drug use for BPH**	Yes	20	62.5	14	46.7	*p* = 0.307
	No	12	37.5	16	53.3	
**Drug use for COPD**	Yes	0	0	2	6.7	*p* = 0.230
	No	32	100	28	93.3	

BPH: Benign prostatic hyperplasia; COPD: Chronic obstructive pulmonary disease.

**Table 3:** Comparison of sexual function before and after the intervention between the groups.

Domains	Intervention Group	Control Group	*p*-Value
	*Before the intervention*		
	Mean (standard deviation)	Mean (standard deviation)	
Erectile function	19.69 (10.38)	18.59 (10.92)	Intervention: 0.68 control: 0. 61
Orgasmic function	7.12 (4.12)	6.83 (4.46)	Intervention: 0.73 control: 0.71
Sexual desire	5.16 (2.05)	5.63 (2.28)	Intervention: 0.31 control: 0.54
Intercourse satisfaction	7.03 (4.22)	7.27 (5.07)	Intervention: 0.41 control: 0.59
Overall satisfaction	6.12 (2.62)	5.73 (2.57)	Intervention: 0.98 control: 0.10
Sexual function	45.12 (22.21)	44.03 (24.34)	Intervention: 0.61 control: 0.54
	*One month after the intervention*		
Erectile function	13.59 (11.03)	8.70 (10.58)	Intervention: 0.195 control: 0.261
Orgasmic function	4 (4)	2.60 (3.82)	Intervention: 0.273 control: 0.519
Sexual desire	5.16 (2.20)	3.63 (2.10)	Intervention: 0.028 control: 0.256
Intercourse satisfaction	4.12 (3.96)	2.67 (3.96)	Intervention: 0.268 control: 0.464
Overall satisfaction	4.84 (2.55)	3.43 (2.22)	Intervention: 0.06 control: 0.35
Sexual function	31.72 (22.38)	21.03 (21.93)	Intervention: 0.15 control: 0.315
	*Three months after the intervention*		
Erectile function	19.09 (10.41)	12.67 (9.78)	Intervention: 0.044 control: 0.374
Orgasmic function	6.28 (3.97)	4.90 (3.85)	Intervention: 0.331 control: 0.318
Sexual desire	5.69 (2.27)	3.93 (2.05)	Intervention: 0.01 control: 0.29
Intercourse satisfaction	6.94 (4.17)	4.27 (3.45)	Intervention: 0.03 control: 0.28
Overall satisfaction	5.57 (2.81)	3.83 (2.16)	Intervention: 0.01 control: 0.61
Sexual function	43.75 (22.62)	29.60 (20.18)	Intervention: 0.038 control: 0.33

**Table 4:** Effects of the intervention and time on the sexual function.

Interaction Effect	*p*-Value
Time	0.92
Time and intervention	0.04
Intervention	0.24
